# Nucleoporin 107 facilitates the nuclear export of *Scn5a* mRNA to regulate cardiac bioelectricity

**DOI:** 10.1111/jcmm.14051

**Published:** 2018-12-03

**Authors:** Yi Guan, Xueting Gao, Qiuyu Tang, Lin Huang, Siyun Gao, Shuai Yu, Jiale Huang, Jun Li, Daizhan Zhou, Yangyang Zhang, Dan Shi, Dandan Liang, Yi Liu, Li Li, Yingyu Cui, Liang Xu, Yi‐Han Chen

**Affiliations:** ^1^ Heart Health Center East Hospital Tongji University School of Medicine Shanghai China; ^2^ Institute of Medical Genetics Tongji University Shanghai China; ^3^ Key Laboratory of Arrhythmias of the Ministry of Education of China East Hospital Tongji University School of Medicine Shanghai China; ^4^ Department of Pathology and Pathophysiology Tongji University School of Medicine Shanghai China

**Keywords:** electrical activity, mRNA export, nucleo‐cytoplasmic transport, Nup107, *Scn5a* mRNA

## Abstract

Nucleoporins (Nups) are known to be functional in nucleo‐cytoplasmic transport, but the roles of nucleoporins in nonproliferating cells, such as cardiac myocytes, are still poorly understood. In this study, we report that Nup107 regulates cardiac bioelectricity by controlling the nucleo‐cytoplasmic trafficking of *Scn5a* mRNA. Overexpression of Nup107 induced the protein expression of *Scn5a* rather than that of other ion channels, with no effects of their mRNA levels. The analysis for the protein production demonstrated Nup107‐facilitated transport of *Scn5a* mRNA. Using RIP‐PCR and luciferase assay, we found that the 5′‐UTR of *Scn5a* mRNA was not involved in the interaction, whereas the spatial interaction between Nup107 protein and *Scn5a* mRNA was formed when *Scn5a* mRNA passing through the nuclear pore. Functionally, Nup107 overexpression in neonatal rat ventricle myocytes significantly increased the currents of *Scn5a*‐encoded I_Na_ channel. Moreover, the close correlation between Nup107 and Nav1.5 protein expression was observed in cardiomycytes and heart tissues subjected to hypoxia and ischaemic insults, suggesting a fast regulation of Nup107 on Nav1.5 channel in cardiac myocytes in a posttranscriptional manner. These findings may provide insights into the emergent control of cardiac electrophysiology through Nup‐mediated modulation of ion channels.

## 1 INTRODUCTION

The nuclear pore complexes (NPCs) span the nuclear envelope, and function as gatekeepers of the nucleus, mediating the exchange of molecules between the nucleoplasm and the cytoplasm.^1^ They contain about 30 different proteins, known as nucleoporins (Nups) to form an eightfold‐symmetrical structure, consisting of a membrane‐embedded scaffold built around a central transport channel, a cytoplasmic ring, a nuclear ring, and eight filaments attached to each ring.^2^ These nucleoporins are organized in multiple subcomplexes around a central eightfold rotational symmetry axis. The outer ring of the NPC scaffold comprises Nup107 complex, the inner ring contains Nup93 complex, and the central transport channel anchors Nup62 complex.^3^, ^4^

The Nup107 complex, the most characterized NPC subcomplex, is comprised of nine members, Nup160, Nup133, Nup107, Nup98/96, Nup85, Nup43, Nup37, Sec13, and Seh1.^5^, ^6^ It is essential for NPC assembly^7^, ^8^ and mRNA export.^9^ The Nup107 complex is also involved in the mitotic process,^10^, ^11^ and regulating microtubule polymerization at kinetochores.^12^ The mutation of Nup107 often leads to developmental abnormality. It has been demonstrated that the disruption of Nup107 in zebrafish embryos causes missing of pharyngeal skeleton, the absence of the swim bladder, and smaller eyes.^13^ The depletion of mouse Nup96 also causes embryonic lethality.^14^ Moreover, the Nup107 mutation is linked to human disease. There is evidence showing that the biallelic NUP107 mutations cause microcephaly and Steroid‐Resistant Nephrotic Syndrome.^15^, ^16^ Our previous study has indicated that Nup107 complex is significantly increased in infarcted myocardial tissues in rat.^17^

In this study, we demonstrated that Nup107 regulates the cardiac electricity by facilitating *Scn5a* mRNA export in cardiomyocytes. The spatial interaction between Nup107 protein and *Scn5a* mRNA is implicated in the regulation of *Scn5a* mRNA export. Furthermore, we showed that Nup107 overexpression was functional in increasing the amplitude of *Scn5a‐*encoded Na+ currents, and associated with ischaemic cardiac injury by increasing the nucleo‐cytoplasmic transport of *Scn5a* mRNA.

## 2 MATERIALS AND METHODS

### 2.1 Ethics approval

Animals in the study were maintained in accordance with the Guide for the Care and Use of Laboratory Animals (NIH Publication). All the experimental procedures with animals used in this study were approved by the Institutional Animal Care and Use Committee of Tongji University School of Medicine.

### 2.2 Experimental myocardial infarction model

Myocardial infarction (MI) was performed in female Sprague Dawley rats as described previously.^18^, ^19^ Briefly, rats were lightly anaesthetized with isoflurane, intubated, and then ventilated with a rodent respirator. The chest cavity was opened via left thoracotomy. Myocardial infarction was induced by ligation of the left anterior descending artery (LAD) with a 6‐0 silk suture at the site of its emergence from the left atrium. The sham‐operated animals underwent the same procedure without LAD ligation. At 72 hours after MI, the surviving animals were killed, and their hearts were quickly excised and rapidly frozen in liquid nitrogen.

### 2.3 Cell culture

Neonatal rat ventricle myocytes (NRVMs) were isolated from hearts of 1‐ to 3‐day‐old Sprague Dawley rats. Cardiomyocytes were cultured in Dulbecco's modified Eagle's medium (DMEM) medium containing 10% foetal bovine serum (FBS) and 100 μmol L^−1^ BrdU for 24 hours and maintained in DMEM containing 10% FBS, 2 mmol L^−1^ L‐glutamine and 1% penicillin/streptomycin (P/S) (Gibco, Invitrogen, Carlsbad, CA, USA). HEK293 cells were cultured and maintained in DMEM with 10% FBS and 1% P/S.

### 2.4 Transfections and plasmids

The full‐length of human Nup107 was PCR‐amplified from the cDNA of HEK293 cells and cloned into pCMV3‐GFPSpark (Sino Biological, Beijing, China) using ClonExpress^®^ II One Step Cloning Kit (C112‐02, Vazyme Biotech Co., Ltd., Nanjing, Jiangsu, China). Different truncates of Nup107 were constructed, including Nup107 full length (FL), Nup107‐N‐terminus (1‐304 aa), Nup107‐conserved domain (CD, 305‐663 aa), Nup107‐C‐terminus (664‐925 aa), Nup107 truncates lacking the N‐terminus (ΔN‐terminus), lacking the conserved domain (ΔCD), or lacking the C‐terminus (ΔC‐terminus), by amplifying the corresponding sequences. The nucleotide sequences of these constructs were confirmed by Sanger sequencing. Transient plasmid transfections were performed with Lipofectamine 3000 (Invitrogen) according to the manufacturer's protocol.

### 2.5 Adenovirus construction and infection

Recombinant adenoviruses expressing green fluorescent protein (Ad‐GFP) and Nup107 (Ad‐Nup107) were prepared using an AdEasy vector system (R&S biotech, Shanghai, China). NRVMs were isolated, cultured, and infected with Ad‐Nup107 and Ad‐GFP adenoviruses (multiplicity of infection = 100).

### 2.6 Quantitative reverse transcription‐polymerase chain reaction (QT‐PCR)

Total RNA was extracted using Trizol reagent (Invitrogen) and reverse transcribed into cDNA using the PrimeScript RT reagent Kit (Takara, Dalian, Liaoning, China). Detection of gene expression was performed with SYBR Green Master Mix and the 7900 Real‐Time PCR System (Applied Biosystems, Foster City, CA, USA). The expression levels of *Scn5a* mRNAs were calculated using the DeltaDeltaCT method, and were normalized against the levels of multiple housekeeping genes (Gapdh, 18s, β‐actin, and β‐Tubulin). All measurements were performed in triplicate. Melting curve analysis confirmed amplification specificity.

For the detection of the mRNA levels in different fractions, RNAs from the cytoplasm and nucleus were isolated according to the manufacturer's instructions (PARIS Kit AM1921, Invitrogen). Each RNA fraction was subsequently reverse‐transcribed using PrimeScript RT reagent Kit (RR036A, Takara, Kyoto, Japan). The absolute quantitative method was used to estimate the relative amount of indicated mRNA in cytoplasm and nucleus. This method is based on the construction of a standard curve of cycle number at a threshold (CT) vs the initial input amount of mRNA copy number. The PCR products containing fragments of the cDNA of each gene were used as the initial input. All the primer sequences are listed in Table S1.

### 2.7 Immunoblots and antibodies

Total proteins were extracted with RIPA buffer (Beyotime, Zhejiang, China) containing protease inhibitor cocktail tablets (Roche Applied Science, Mannheim, Germany), and separated by SDS‐PAGE (Invitrogen). Then they were transferred onto PVDF membranes and probed with antibodies against Nav1.5, Kv4.3, and Cav1.2 (ASC‐005, APC‐017, and ACC‐003, Alomone Labs, Jerusalem, Israel), Kir2.1 and Kv1.4 (19965‐1‐AP, and 19697‐1‐AP, ProteinTech Group, Wuhan, Hubei, China), Nup107 and GFP (ab73290, and ab290, Abcam, Cambridge, MA, USA), β‐actin, and GAPDH (66009‐1‐Ig and 60004‐1‐Ig, ProteinTech Group). After washing, blots were treated with the appropriate IRDye 800 conjugated secondary antibody (072‐07‐15‐06 and 072‐07‐18‐06, LI‐COR Biosciences, Lincoln, NE, USA) at room temperature for 1 hours. Images were recorded using the Odyssey infrared imaging system and analysed using the Odyssey Application Software v2 (LI‐COR Biosciences).

### 2.8 Luciferase reporter assay

For the functional analysis of *Scn5a* mRNA, the luciferase reporter vectors harbouring different parts of *Scn5a* mRNA (5′‐untranslated region (UTR), coding sequence (CDS), and 3′‐UTR) were constructed as follows: the sequence of 5′‐UTR of rat *Scn5a* mRNA (NM_013125) was synthesized and inserted into the 5′ NHE I of the Renilla luciferase gene in psiCheck2 (Promega, Madison, WI, USA). The different regions of the 3′‐UTR of *Scn5a* mRNA (NM_013125; full length: +1~+2193, +1~+1000, and +1001~+2193) were amplified from the cDNA of H9c2 cells and cloned into the 3′ downstream of the Renilla luciferase gene using the Xho I and Not I sites. The CDS regions of human *SCN5A* mRNA (NM_198056; full length: 1~6051, 1~3000, and 3001~6051) were amplified from pCMV6‐*SCN5A*‐GFP (RG214786, Origene Technologies, Rockville, MD, USA) and cloned into the Xho I and Not I sites of psiCheck2 vector. The resultant psiCheck2 constructs were cotransfected with Nup107 and its truncates, or control vector in HEK293 cells for 48 hours. Afterwards the firefly and renilla luciferase activities were analysed using the Dual‐Luciferase Reporter Assay System (Promega).

### 2.9 RNA immunoprecipitation (RIP)

The RIP experiment was performed using a Millipore EZ‐Magna RIP RNA‐Binding Protein Immunoprecipitation kit (17‐701, Millipore, Bedford, MA, USA) according to the manufacturer's instructions. NRVMs were infected with Ad‐Nup107 or Ad‐GFP for 48 hours and harvested for the endogenous *Scn5a* mRNA immunoprecipitation. Antibodies used for RIP included rabbit polyclonal IgG (Millipore, PP64), GFP, and Nup107 (ab290 and ab73290, Abcam), and 5 μg of antibody was used per RIP reaction. The immunoprecipitated RNA was extracted with Trizol and reverse‐transcribed with PrimeScript RT reagent Kit (Takara). All RIP assays were performed with biological duplicates. The RIP‐PCR was used to measure the amount of *Scn5a* mRNA in pull‐down samples. The data were demonstrated as the percentage of input GAPDH mRNA of its respective group. P values were obtained using two‐tailed Student's *t*‐test.

### 2.10 Electrophysiological recording

Whole‐cell patch‐clamp recordings were performed at room temperature (24°C) using an EPC‐10 amplifier and pulse software (HEKA, Ludwigshafen, Germany) as previous reports.^20^, ^21^ I_Na_ was recorded with NRVMs bathed in a solution containing (in mmol L^−1^): 20 NaCl, 50 TEA‐Cl, 67 CsCl, 1 MgCl_2_, 1 CaCl_2_, 0.1 CdCl_2_, 10 glucose and 10 Hepes, pH 7.4 with CsOH. Pipette solution consisted of (in mol L^−1^) 5 NaCl, 125 CsF, 10 EGTA, 10 Hepes and 5 Mg‐ATP,pH 7.2 with CsOH. Cells were held at −120 mV and I_Na_ was elicited by a family of voltage steps to potentials ranging from −70 to +40 mV with 10 mV increments for 50 ms, with 5000 ms interpulse intervals.

### 2.11 Statistical analysis

All data are presented as the mean ± SD. Statistical comparisons among multiple groups were performed by ANOVA and unpaired Student's *t*‐test, as appropriate. The results were considered statistically significant if *P* < 0.05.

## 3 RESULTS

### 3.1 Nup107 selectively increases the expression of Nav1.5 protein in cardiomyocytes

**Figure 1:**
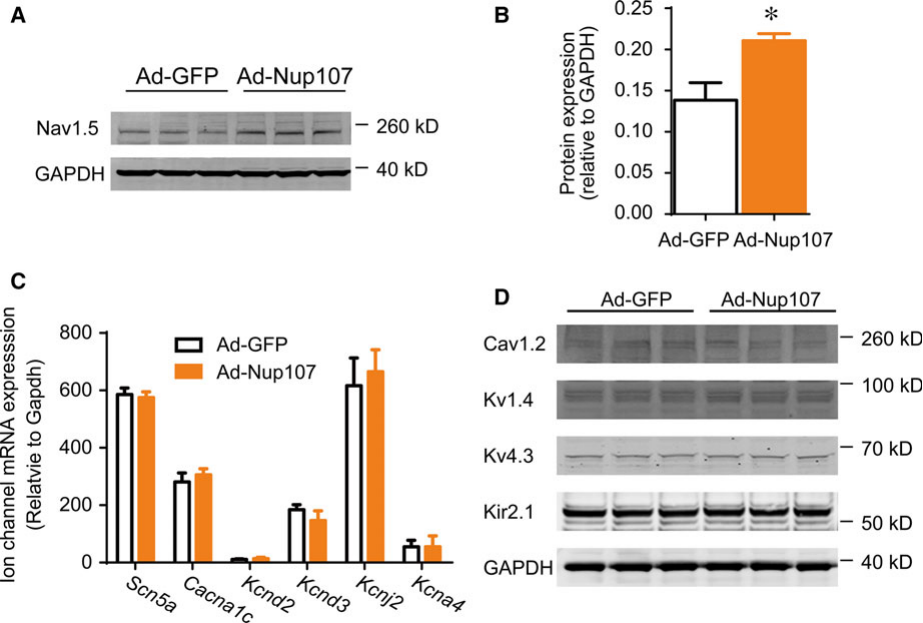
Overexpression of Nup107 increases Nav1.5 protein expression in cardiomyocytes. (A) Western blotting examination of Nup107 overexpression on Nav1.5 protein expression. NRVMs were treated with either control (Ad‐GFP) or Ad‐Nup107 for 48 h, followed by the detection of Nav1.5 protein expression. (B) Data were pooled from three independent experiments. (C) Nup107 overexpression did not affect the mRNA levels of major ion channels. NRVMs were infected with Ad‐GFP or Ad‐Nup107 for 48 h, followed by the RT‐PCR analysis of sodium channel (*Scn5a*), calcium channel (*Cacna1c*), and potassium channels (*Kcnd2*,* Kcnd3*,* Kcnj2,* and *Kcna4*). (D)Western blotting examination of Nup107 overexpression on protein expression of other ion channels. NRVMs were treated with either Ad‐GFP or Ad‐Nup107 for 48 h, and then subjected to the detection of calcium channel (Cav1.2), and potassium channel associated proteins (Kv4.3, Kv1.4, and Kir2.1)

Our previous study has revealed the dynamic expression of nucleoporins in infarcted myocardial tissues in rat (Supplementary materials, Figure S15).^17^ To identify the common nucleoporins differentially expressed in impaired heart tissues, we further examined mouse myocardial samples from left ventricles 3 days after myocardial infarction. Interestingly the mRNA level of Nup107 was significantly increased, consistent with our data mining from previous report (22, GSE83350) (Figure S1). As Nup107 is the core member of Nup107 complex in the nuclear protein complex (NPC), and its anomaly is associated with numerous physiological and pathological process, we focused on the function of Nup107 in the ischaemic cardiac injury. To better understand Nup107 functions in NRVMs, we applied adenovirus‐mediated Nup107 overexpression. The overexpression efficiency of Nup107 was evaluated with fluorescent imaging and Western blotting (Figure S2). Considering that Nup107 mRNA was significantly increased 3 days after myocardial infarction, when is concomitant with the susceptible period of cardiac arrhythmia, we attempted to explore the potential roles of Nup107 in the regulation of ion channel expression. As shown in Figure 1A and B, the overexpression of Nup107 significantly increased the expression of *Scn5a*‐encoded Nav1.5 protein, whereas other ionic channels, such as calcium channel (*Cacna1c*) and potassium channels (*Kcnd2*,* Kcnd3*,* Kcnj2,* and *Kcna4*) were not affected in both mRNA and protein expression (Figure 1C and D). These findings suggest that Nup107 is an important regulator for Nav1.5 protein expression.

### 3.2 Nup107 facilitates mRNA export to increase Nav1.5 protein in cardiomyocytes

**Figure 2:**
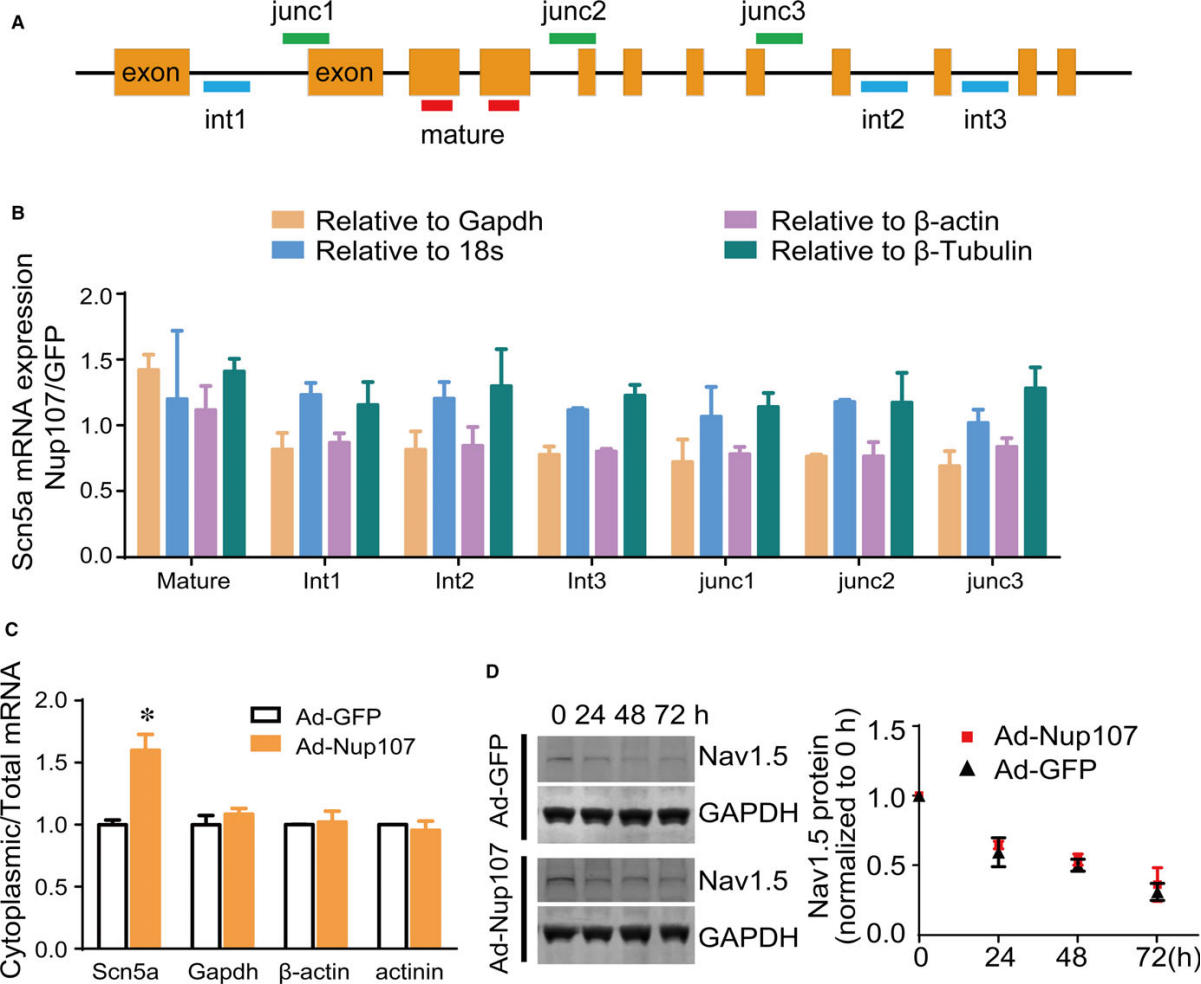
Overexpression of Nup107 changes the distribution of *Scn5a* mRNA between the cytoplasm and nucleus in NRVMs. (A) Schematic diagram of amplification regions of mature and nascent *Scn5a* mRNA. Junc, junction region; int, intron region. (B) Effects of Nup107 overexpression on the expression of mature and nascent *Scn5a* mRNA. The expression of *Scn5a* mRNA was normalized to multiple internal reference Gapdh, 18s, β‐actin, and β‐tubulin. Data were pooled from three independent experiments. (C) Overexpression of Nup107 altered the cytoplasmic‐nuclear distribution of *Scn5a* mRNA in NRVMs. The copies of Scn5a, Gapdh, β‐actin, and actinin mRNA in cytoplasmic and nuclear fractions were measured using absolute qualification. Data were expressed as the ratio of cytoplasmic and total mRNAs, and pooled from three independent experiments. *P < 0.01. (D) The half‐life of Nav1.5 was not affected in NRVMs, as determined by a cycloheximide (CHX) chase assay. NRVMs were infected with either control Ad‐GFP or Ad‐Nup107 for 24 h, followed by the addition of CHX (100 μg/mL) for the indicated time. Then the cells were harvested and immunoblotted. *Left*, typical blots of Nav1.5 expression in Nup107 overexpressed NRVMs; *right*, pooled data of Nav1.5 expression in three independent experiments

The expression level of a protein depends on the rate of transcription, translation, transportation, and degradation. To gain insight into the increment in Nav1.5 protein by Nup107 overexpression, we first analysed the transcript levels of *Scn5a* mRNA. We designed the primers that annealed to the intron (int), or the junction region spanning the junction (junc) of the intron and exon to detect the unspliced *Scn5a* mRNA, or the exon‐spanning primers that detected only the spliced, mature *Scn5a* mRNA. To exclude the possibility that the overexpression of Nup107 could affect the expression of internal references, multiple genes, such as GAPDH, β‐actin, β‐tubulin, and 18s, were used to normalize the mRNA level of *Scn5a* mRNA. Our data showed that Ad‐Nup107 infection did not affect the production of either nascent or mature *Scn5a* mRNA in NRVMs, compared with the Ad‐GFP‐treated cells (Figure 2A and B). On the basis of the presence of Nup107 on the nuclear envelope (NE), we asked whether the transport of *Scn5a* mRNA from nucleus to cytoplasm was responsible for the Nav1.5 protein increase in Nup107 overexpressed NRVMs. Therefore, the RNAs were isolated from cytosolic and nuclear fractions of adenovirus infected NRVMs and measured using qRT‐PCR. As shown in Figure 2C, Nup107 overexpression for 48 hours significantly increased the cytoplasmic ratio of *Scn5a* mRNA, but not of Gapdh, β‐actin, and cardiac specific ɑ‐actinin. Next we tested the potential contribution of protein degradation in Nup107‐mediated regulation of Nav1.5 protein. We found that the application of cycloheximide (CHX, a specific inhibitor of protein synthesis) did not alter the lifespan of Nav1.5 protein in Nup107‐overexpressed NRVMs (Figure 2D). Taken together, these results suggest that the Nup107‐mediated regulation of *Scn5a* expression is involved in the nuclear export of *Scn5a* mRNA without the effects of protein degradation and mRNA synthesis.

### 3.3 The spatial interaction between Nup107 protein and *Scn5a* mRNA is critically required for *Scn5a* posttranscriptional regulation in cardiomyocytes

**Figure 3:**
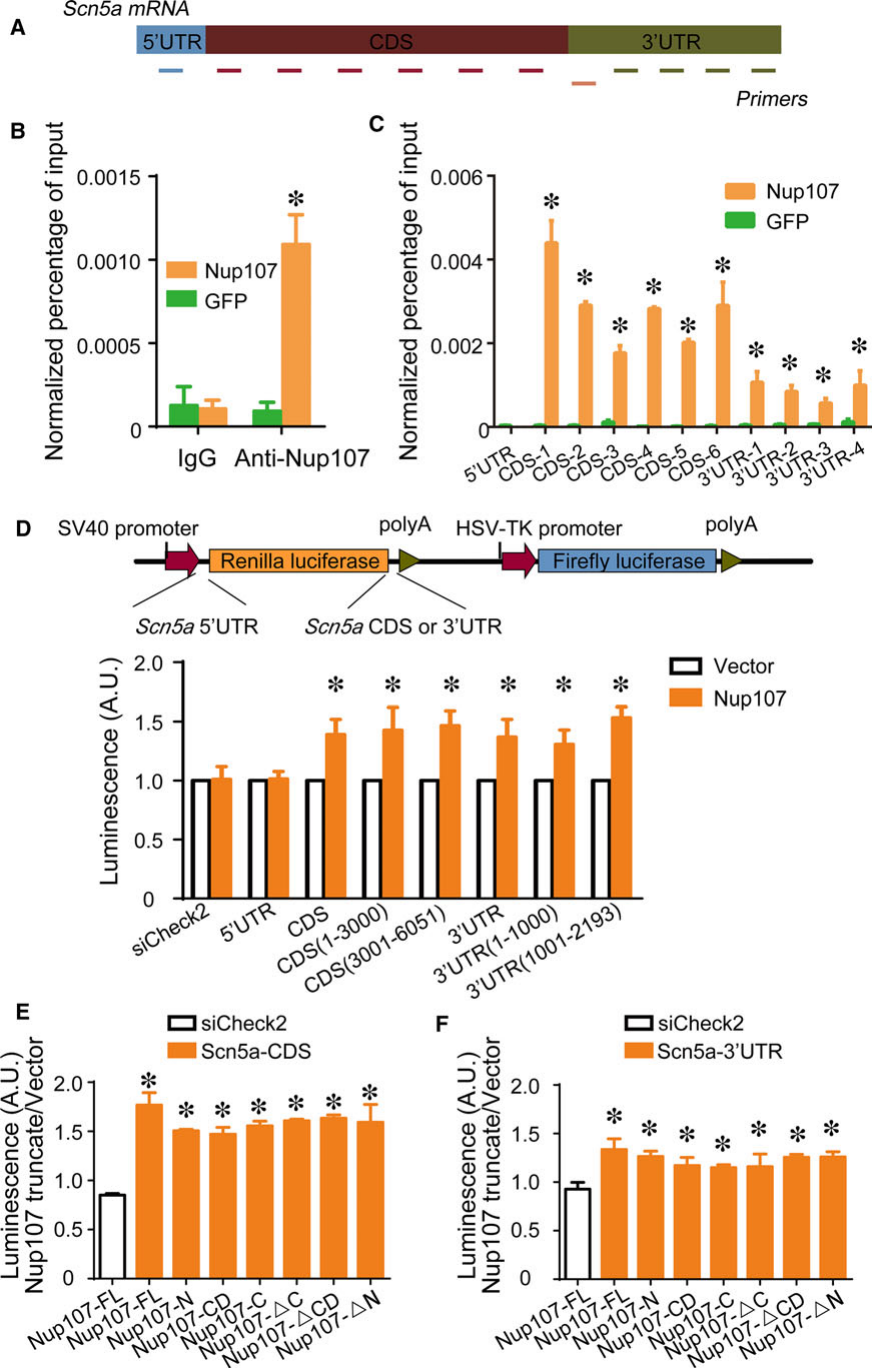
Identification of interactive sites for Nup107 and *Scn5a* mRNA. (A) Schematic diagram of primer pairs specific for different parts of *Scna5a* mRNA. UTR, untranslated region; CDS, coding sequences. (B) RNA‐IP assay for detecting the interaction between Nup107 protein and *Scn5a* mRNA. NRVMs were treated with Ad‐Nup107 or Ad‐GFP for 48 h, followed by RNA‐IP assay. The RIP‐PCR analysis showed that Nup107, but not GFP selectively regulated *Scn5a* mRNA. *P < 0.01. (C) Association of Nup107 with different regions of *Scn5a* mRNA in NRVMs as measured by RIP‐PCR analysis. Values are the means ± SD (n = 3). *P < 0.01 compared with GFP control group. (D) The interactive regions of *Scn5a* mRNA associated with Nup107 protein, verified by the luciferase reporter assay. *Top*, schematic diagram of the luciferase reporter vectors containing the 5′‐UTR, CDS, or 3′‐UTR of *Scn5a* mRNA;* bottom*, the luminescence intensity under the overexpression of Nup107 and control vector transfected with the 5′‐UTR, CDS, 3′‐UTR reporters, or their corresponding subcloning reporters in HEK293 cells. HEK293 cells overexpressed with Nup107 were cotransfected with the luminescence vectors containing the 5′‐UTR, CDS, or 3′‐UTR of *Scn5a* mRNA and their subcloning reporters (CDS (1‐3000, and 3001‐6051); 3′‐UTR (1‐1000, and 1001‐2193)) for another 48 h. The data of the luminescence intensity were presented as the mean ± SD of three independent experiments. *P < 0.01. The luminescence intensity under the overexpression of Nup107 and its truncates transfected with the *Scn5a*‐CDS (E) or 3′‐UTR (F) reporters in HEK293 cells. HEK293 cells ectopically expressed with *Scn5a*‐CDS or *Scn5a*‐3′‐UTR reporters were cotransfected with Nup107 full‐length and its truncates (the N‐terminus, the C‐terminus, the conserved domain (CD),the constructs lacking the N‐terminus (ΔN), lacing the C‐terminus (ΔC), and lacking CD (ΔCD)) for 48 h. The data of the luminescence intensity were presented as the mean ± SD of three independent experiments. *P < 0.01

To investigate how Nup107 regulated the nuclear export of *Scn5a* mRNA, the RNA‐IP experiment was performed to identify the potential interactive sites on *Scn5a* mRNA. The Nup107 proteins were immunoprecipitated from NRVMs infected with Ad‐Nup107, followed by RT‐PCR analysis of their binding mRNAs. The primer pairs specific for different regions of *Scn5a* mRNA including 5′‐untranslated region (UTR), coding DNA sequence (CDS), and 3′‐UTR, were used to identify the potential regions interactive with Nup107 (Figure 3A). Remarkably, *Scn5a* mRNA could be coimmunoprecipitated with Nup107 protein in cardiac myocytes, compared with GFP group (Figure 3B). Interestingly, significant interaction was detected in both CDS and 3′‐UTR regions, but not in the 5′‐UTR of *Scn5a* mRNA (Figure 3C), suggesting that a secondary structure of *Scn5a* mRNA is formed for the interaction with Nup107 protein. To verify the binding regions of *Scn5a* mRNA for Nup107‐mediated nuclear export, we cloned the 5′‐UTR, 3′‐UTR, and CDS of *Scn5a* mRNA and inserted them into the 5′ or 3′ position of the CDS of luciferase to compare the relative expression in HEK293 cells (Figure 3D). Our data showed that the ectopic Nup107 significantly increased the intensities of the luminescence in the CDS and 3′‐UTR groups, but had no effects on the 5′‐UTR group. Furthermore, the luminescent reporters containing subcloning segments of CDS and 3′‐UTR of *Scn5a* mRNA exhibited the similar results to their corresponding full‐length reporters (Figure 3D), indicating that the regions of CDS and 3′‐UTR of *Scn5a* mRNA, were critical for the Nup107‐mediated *Scn5a* mRNA export, whereas the 5′‐UTR seemed dispensable for the interaction of *Scn5a* mRNA with Nup107.

Human Nup107 contains an N‐terminal region (aa 1‐304), an Nup84p‐homologous conserved domain (CD) (aa 305‐664) and a C‐terminal region (aa 665‐925). To identify the critical domain responsible for *Scn5a* mRNA export, different truncated mutants of Nup107 were constructed (Figure S3) and coexpressed with luminescent reporters harbouring 3′‐UTR or CDS of *Scn5a* mRNA in HEK293 cells. Unexpectedly, while the Nup107 full‐length overexpression induced an increase in the luminescent intensities of *Scn5a*‐CDS and ‐3′UTR reporters, all the other truncates of Nup107 displayed similar elevation in the *Scn5a*‐CDS and ‐3′UTR luminescent vectors (Figure 3E and F). These data indicate that all the domains of Nup107 are critical for the interaction with *Scn5a* mRNA.

### 3.4 Nup107 increases the amplitude of I_Na_ currents and its upregulation is implicated in pathological cardiac injury

**Figure 4:**
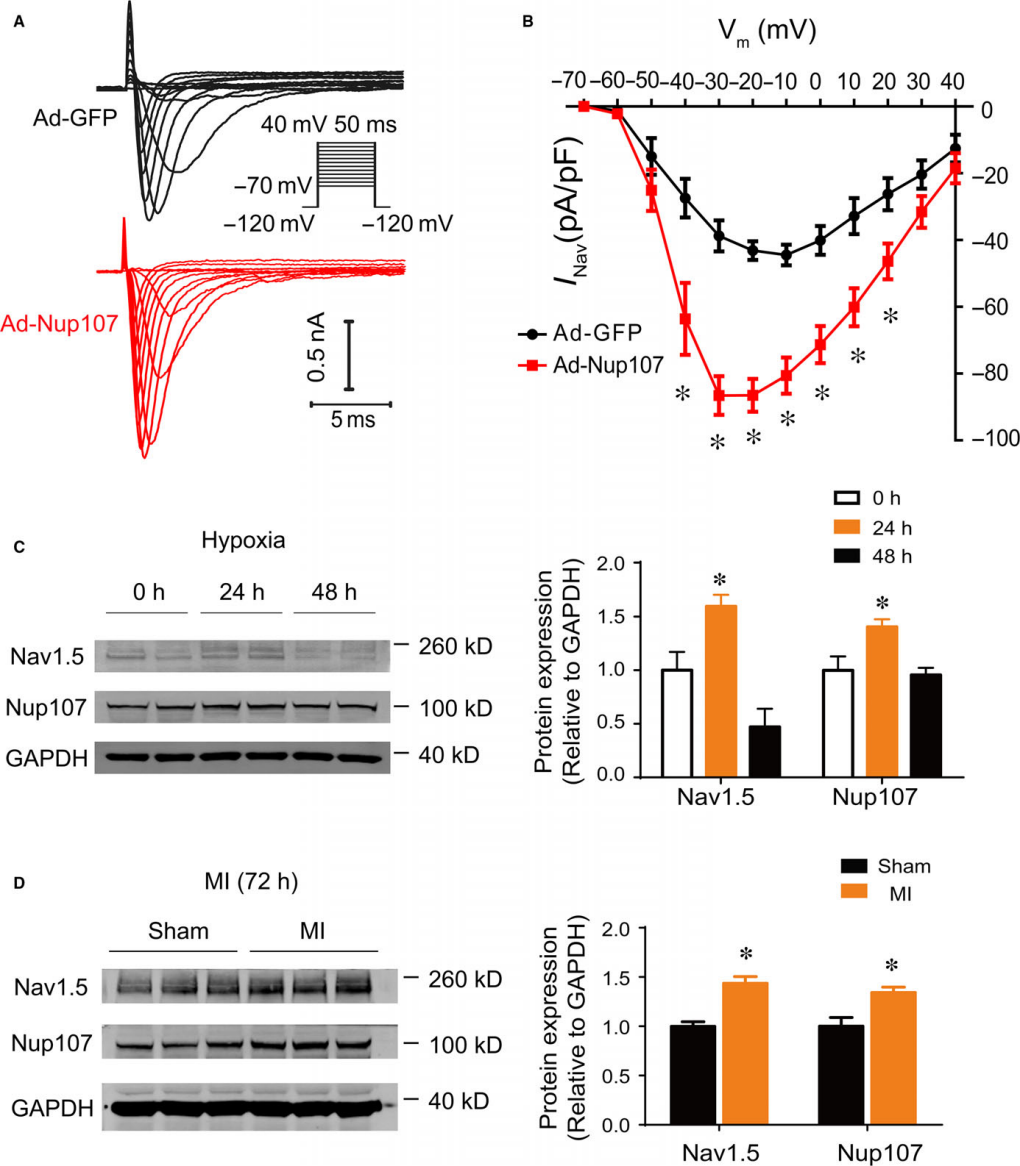
Nup107 regulates cardiac I_Na_ currents and Nup107‐mediated regulation of Nav1.5 is associated with ischaemic cardiac injury both in vitro and in vivo. (A) The representative Na+ currents recorded from NRVMs infected with Ad‐Nup107 or Ad‐GFP. The currents were recorded with the whole‐cell patch‐clamp technique. Cells were held at −120 mV and I_Na_ was elicited by a family of voltage steps to potentials ranging from −70 to +40 mV with 10 mV increments. (B) Current‐voltage relationship of I_Na_ in NRVMs infected with Ad‐Nup107 (n = 20) or Ad‐GFP (n = 12). Currents were normalized to cell capacity. *P < 0.01. (C) Protein expression levels of Nup107 and Nav1.5 in cultured NVRMs subjected to hypoxia condition (1% O_2_) for 48 h. Data are presented as mean ± SD of three independent experiments. *P < 0.01. (D) The protein expression of Nup107 and Nav1.5 in ischaemic ventricular tissues from the rat model of acute myocardial infarction. *Left*: representative blots; *right*: pooled data. *P < 0.01

To determine whether Nup107 could affect the function of Nav1.5 channel, we did the whole‐cell patch‐clamping recordings for the Nup107‐mediated electrophysiological regulation. Our data demonstrated that the current density of the voltage‐gated Nav1.5 channel was dramatically increased in adenovirus‐mediated overexpression of Nup107 in NRVMs (Figure 4A and B). The average of peak inward Na+ current in Nup107‐overexpression group exhibited greater value (−20 mV, 83.87 ± 14.03 pA pF^−1^, n = 20), compared with the control Ad‐GFP group (43.16 ± 7.82 pA pF^−1^ at −20 mV, n = 12, *P* < 0.01). These data indicate that Nup107 overexpression can increase the amplitude of Nav1.5 channel currents in cardiac myocytes.

We next explored the potential contribution of Nup107‐related Nav1.5 expression in pathological heart tissues. Following the development of hypoxia, the protein levels of Nup107 and Nav1.5 were both upregulated at 24 hours, and gradually downregulated at 48 hours after 1% O_2_ treatment (Figure 4C). The application of H_2_O_2_ (100 μmol L^−1^) resulted in marked increase in Nup107 and Nav1.5 proteins in NRVMs (Figure S4). These data suggest that Nup107 could function as a reactive protein, quickly responding to the oxygen alteration. Furthermore, we examined the expression of Nup107 and Nav1.5 in a rat model of acute myocardial infarction. Strikingly, a positive correlation between the protein expression of Nup107 and Nav1.5 was observed at 72 hours after MI in the rat model (Figure 4D), which was similar to that in hypoxia‐induced NRVMs at 48 hours suggesting that the Nup107‐dependent regulation of *Scn5a* may also be relevant to ischaemic cardiac diseases in vivo.

## 4 DISCUSSION

This study provides multiple lines of evidence implicating the role of Nup107 in regulating *Scn5a* mRNA nuclear export and *Scn5a*‐encoded Nav1.5 channel in cardiomyocytes. First, Nup107 specifically regulates the distribution of *Scn5a* mRNA in a posttranscriptional manner. Second, active interaction between Nup107 protein and *Scn5a* mRNA is required for the *Scn5a* mRNA export and subsequent translation. Third, Nup107 increment is functional in regulating cardiac electricity in that its overexpression increases the current densities of *Scn5a*‐encoded Nav1.5 channel. Fourth, Nup107‐mediated regulation of *Scn5a*‐encoded Nav1.5 channel is implicated in hypoxia‐induced myocytes and ischaemic cardiac tissues. These findings suggest that the Nup107‐dependent export of *Scn5a* mRNA is a critical step in regulating cardiac electrical activity.

The Nup107 complex is known to be a pivotal player in NPC formation. It is located in the central scaffold of the NPC and mediates mRNA export in interphase, and has roles in kinetochore function, spindle assembly, and postmitotic nuclear pore assembly. Data mining 22 and our results indicate that the members of Nup107 complex are greatly increased after myocardial infarction, suggesting the dynamical regulation of Nup107 complex in response to cardiac injury.

Our data showed that Nup107 specifically regulated the expression of *Scn5a*, but not other ion channel genes, such as calcium and potassium channels. The regulation occurred in the transportation stage, in which the mature *Scn5a* mRNA was exported from nucleus to cytoplasm. This is consistent with the localization of Nup107 in the NE,^23^, ^24^ where the nucleoporin mediates the communication between cytoplasm and nuclei, without the alteration of mRNA expression.

Till now we do not know the detail of this posttranscriptional regulation, but it seems that the three‐dimensional shape of *Scn5a* mRNA is formed before its entry into the cytoplasm. Interestingly, the 5′‐UTR, which is demonstrated to be a crucial part, guiding the mRNA through the nuclear pore,^17^ has no interaction with Nup107, whereas the CDS and 3′‐UTR of *Scn5a* mRNA are all involved in the contact with Nup107 (Figure 3D). No defined region was identified to be responsible for *Scn5a* mRNA export. As one of the largest mRNAs, the formation of this conformational *Scn5a* mRNA may be necessary and a rate‐limiting step for mRNA export.

Mammalian Nup107 is composed of an N‐terminal region, a C‐terminal region and an Nup84p‐like conserved domain, which is the homologous domain of Nup84p in yeast. Unexpectedly no special domain of Nup107 was found to be critical for the Nup107 protein‐*Scn5a* mRNA interaction (Figure 3E and F). It seems that all the domains of Nup107 participate in the binding of *Scn5a* mRNA. Given that *Scn5a* mRNA forms a three‐dimensional structure before its nuclear export, it is understandable that this conformation of *Scn5a* mRNA is induced by the flexible Nup107, and consequently the interaction between Nup107 protein and *Scn5a* mRNA is dynamically changed in the process of mRNA passing through the nuclear pore. We do not know exactly whether the FG repeats play a role in this reciprocal action, despite Nup107 contains several FG repeats, but the truncated mutant of Nup107 lacking the FG repeats exhibits the similar results to the full‐length of Nup107, indicating the dispensable role of FG repeats in the translocation of *Scn5a* mRNA.

It has been reported that Nup107 forms the Nup107‐160 subcomplex with a number of other nucleoporins^25^, ^26^ and is involved in the assembly of NPC. Herein we cannot exclude the possibility that Nup107‐mediated translocation of *Scn5a* mRNA is regulated indirectly by other nucleoporins, for example, by a complex containing multiple types of nucleoporins.^27^ Our additional experiment indicates that the Nup107 complex seems to take part in the translocation of *Scn5a* mRNA (Figure S5), and the larger members of Nup107 complex appear to be coordinately functional.

Nav1.5, encoded by *Scn5a* gene, is an integral membrane protein. As the pore forming ɑ‐subunit of the voltage‐dependent cardiac Na+ channel, it is fundamental in the initiation and conduction of action potentials in cardiac myocytes.^28^ Our identification of a strong correlation between Nup107 and Nav1.5 both *in vitro* and *in vivo* underlies the regulation of *Scn5a* mRNA by Nup107 under pathological condition. Importantly, the increased expression of Nup107 was observed both in hypoxia and oxidative stress in cardiomyocytes, suggesting that Nup107 is a fast reactive protein responding to the insults. This may represent an emergent responsive mechanism, when the cardiac myocytes encounter the impaired environment.^29^ Compared to the mitotic cell, the cardiac myocyte maybe relies heavily on this reaction, when it cannot repair the tissue by proliferation, and have no time to initiate the transcriptional procedure. Of note, in the process of hypoxia, the protein levels of Nup107 and Nav1.5 are increased in parallel at 24 hours, but at 48 hours, whereas Nup107 maintains its increased protein expression, the Nav1.5 expression dropped, suggesting the exhaust of the existing pool of *Scn5a* mRNA. This is supported by the observation of MI model showing the increased Nup107 and Nav1.5 expression at 72 hours, and increasing Nup107 protein and decreased Nav1.5 expression at 1 week after MI (Figure S6). The 72 hours post‐MI is an important time point involving the dramatic inflammatory process 30 and metabolic alteration,^31^ which represent an immediate response to cardiac injury. Notably, while some of the Nup107 complex members were altered, there were several other nucleoporins, such as Nup153, Nup205, and Nup155, that were changed as well. The dynamic changes of these nucleoporins were found in the time‐course from 3 day to 7 day post‐MI, suggesting their potential roles in the pathological environment. Collectively, these findings convincingly indicate that Nup107 may serve as a novel molecular target for ameliorating myocardial ischaemic injury.

In summary, our findings clarify for the first time the mechanism by which Nup107 regulates cardiac electrical activity through controlling posttranscriptional transportation, and has provided valuable insight into the molecular modulator in cardiac diseases.

## CONFLICT OF INTEREST STATEMENT

The authors confirm that there are no conflicts of interest.
