# Supplementary figures and images for: Nucleoporin 107 facilitates the nuclear export of Scn5a mRNA to regulate cardiac bioelectricity
Source: J Cell Mol Med. 2018 Dec 3;23(2):1448–57. doi: 10.1111/jcmm.14051 (PMC6349201; doi:10.1111/jcmm.14051)

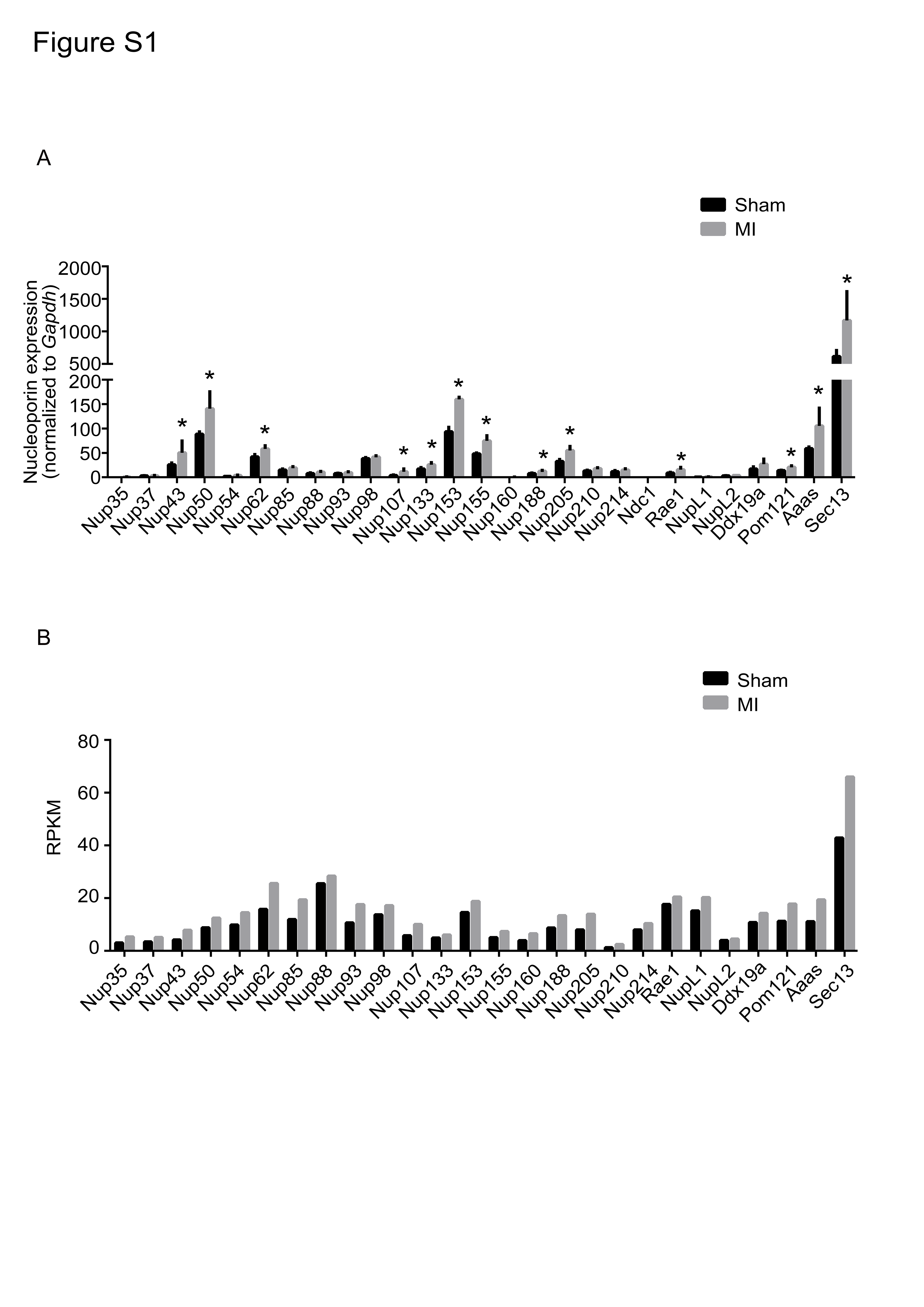

Supplement: Supplementary file 1 [file JCMM-23-1448-s001.tif]

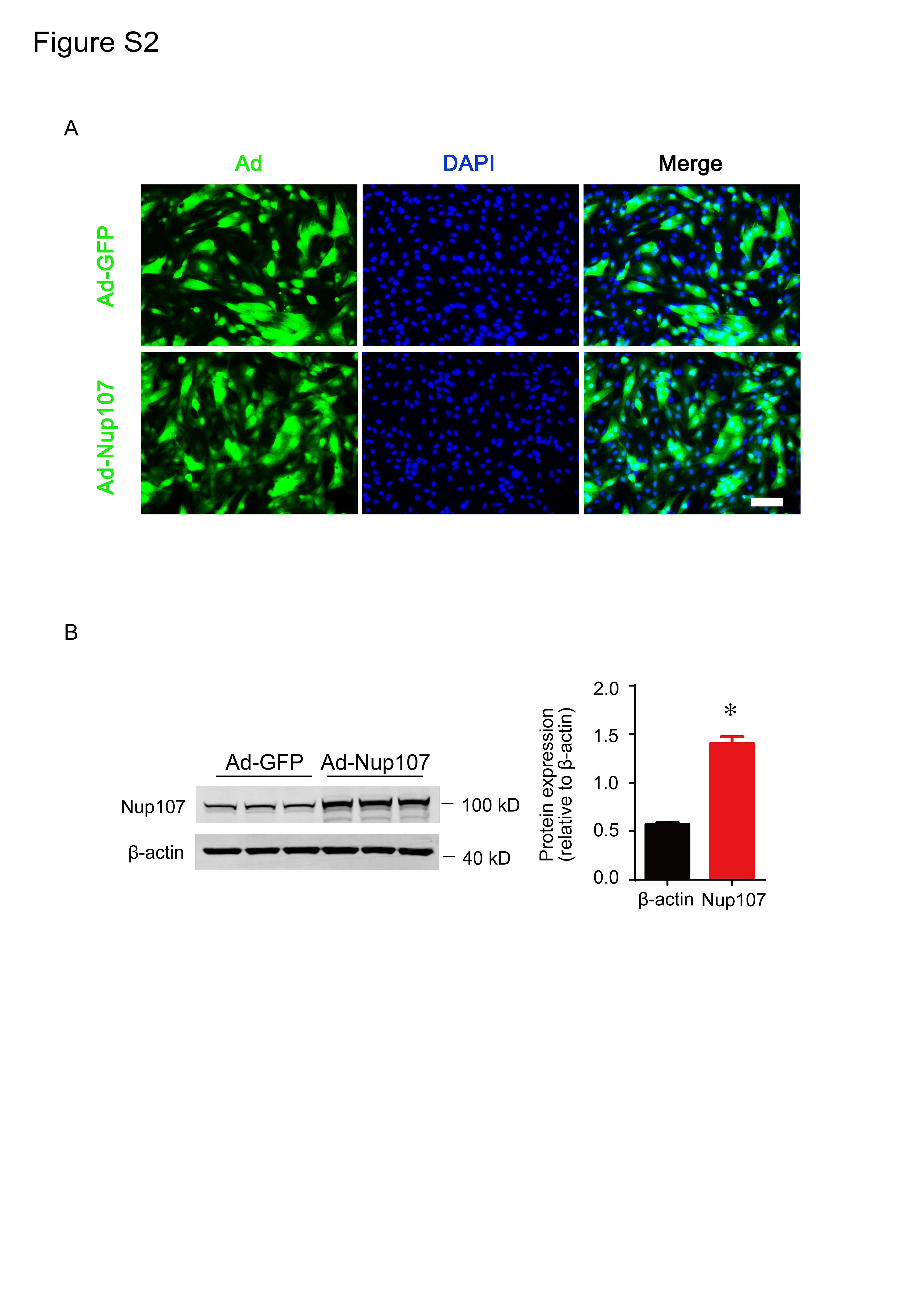

Supplement: Supplementary file 2 [file JCMM-23-1448-s002.tif]

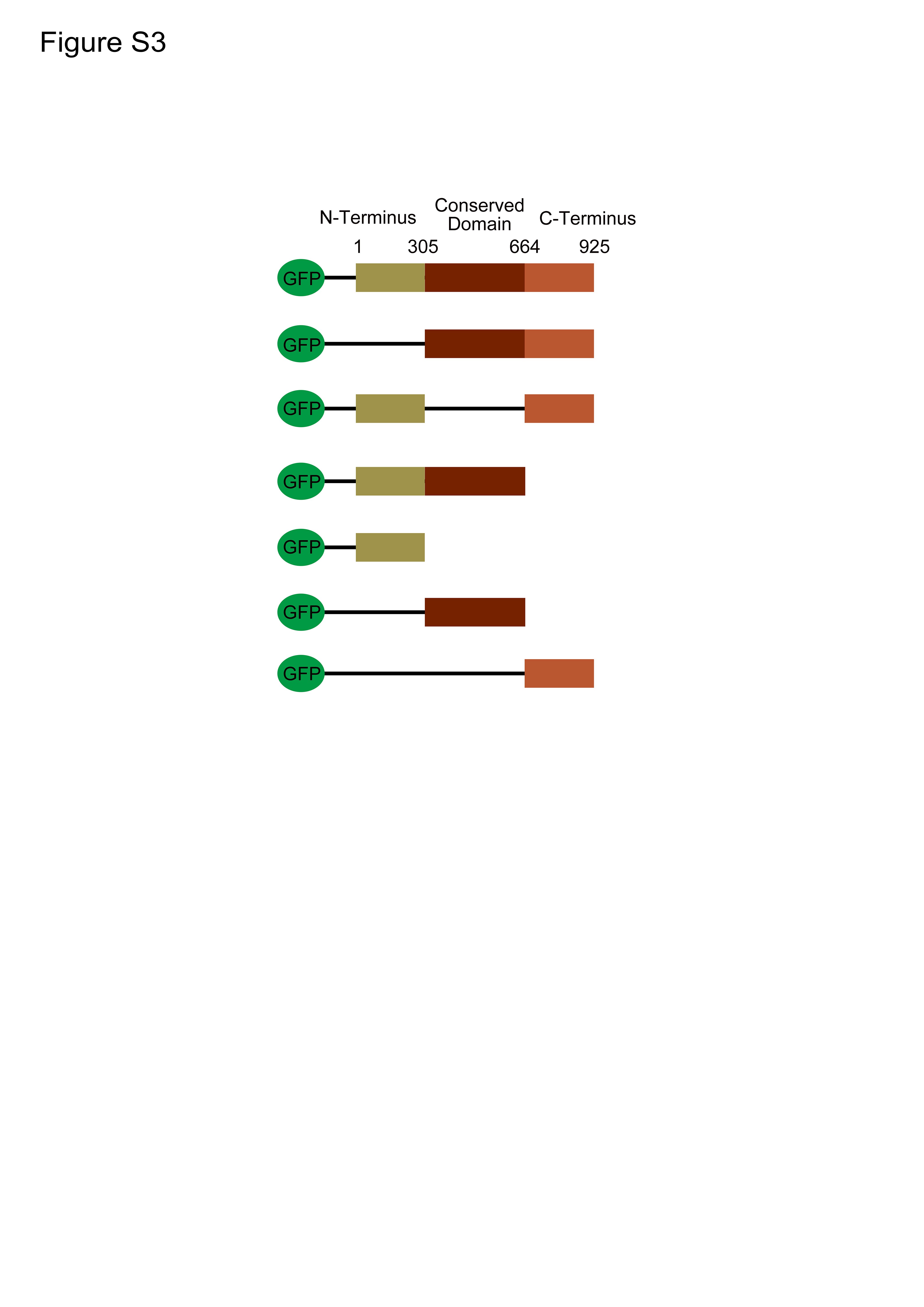

Supplement: Supplementary file 3 [file JCMM-23-1448-s003.tif]

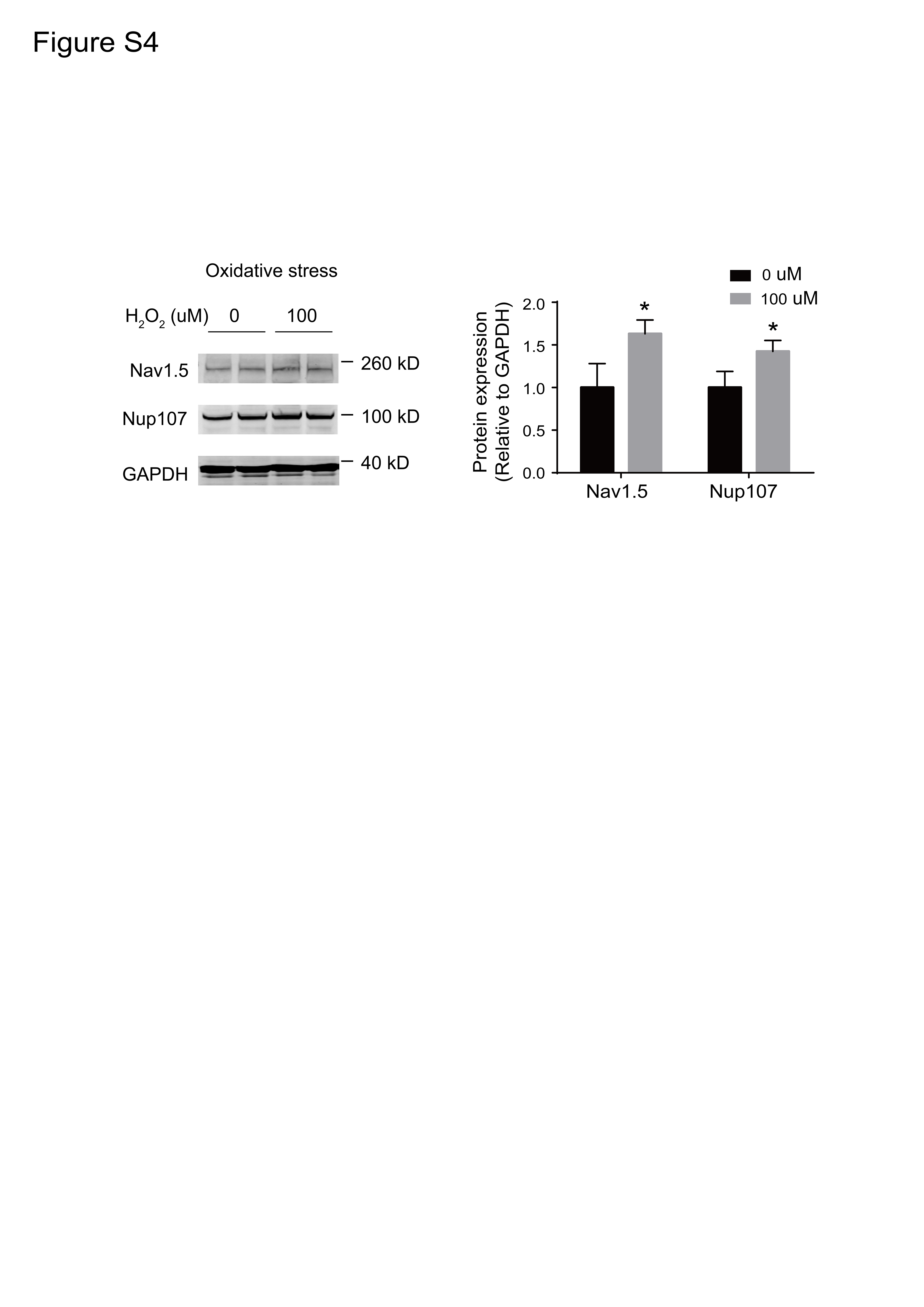

Supplement: Supplementary file 4 [file JCMM-23-1448-s004.tif]

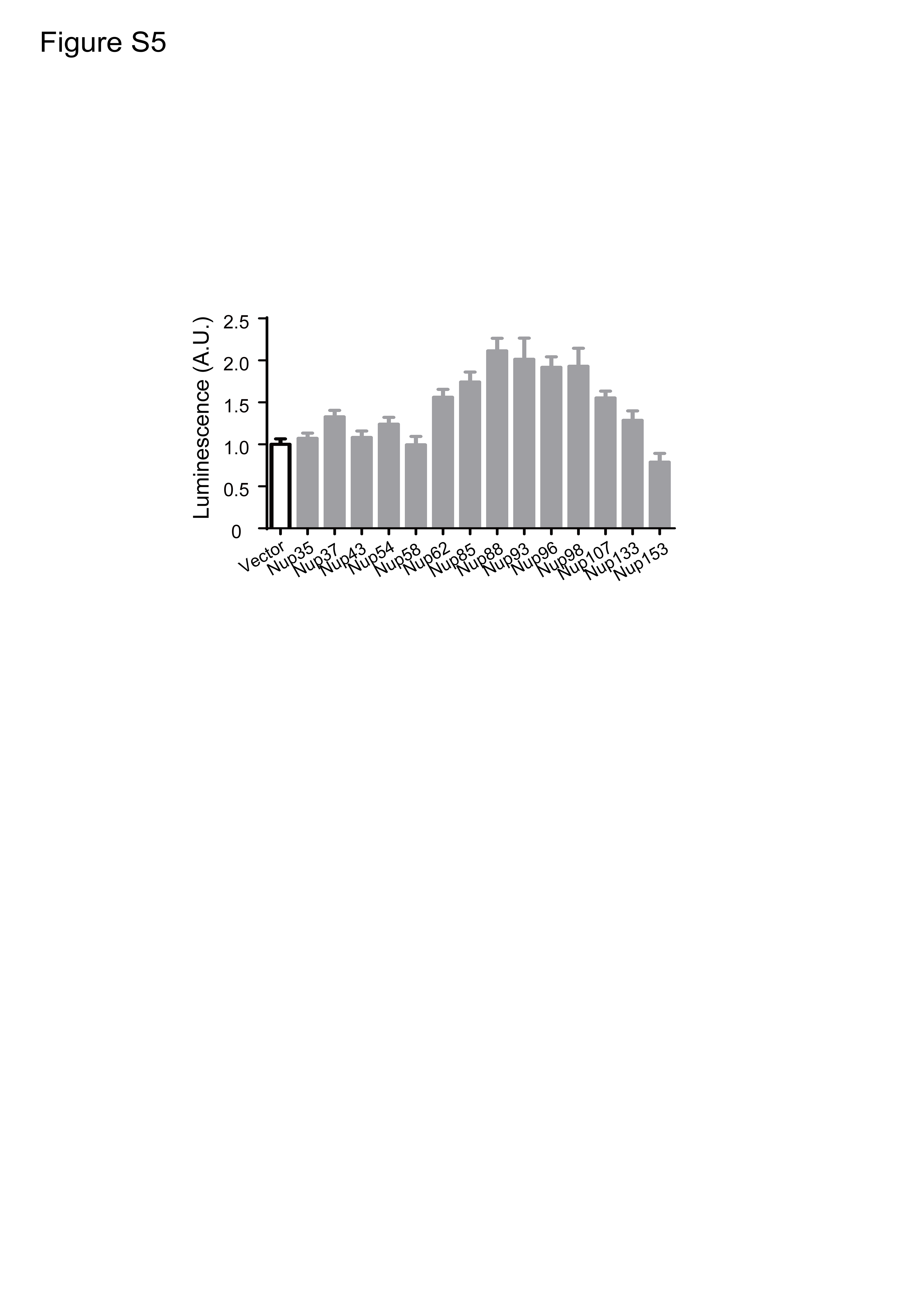

Supplement: Supplementary file 5 [file JCMM-23-1448-s005.tif]

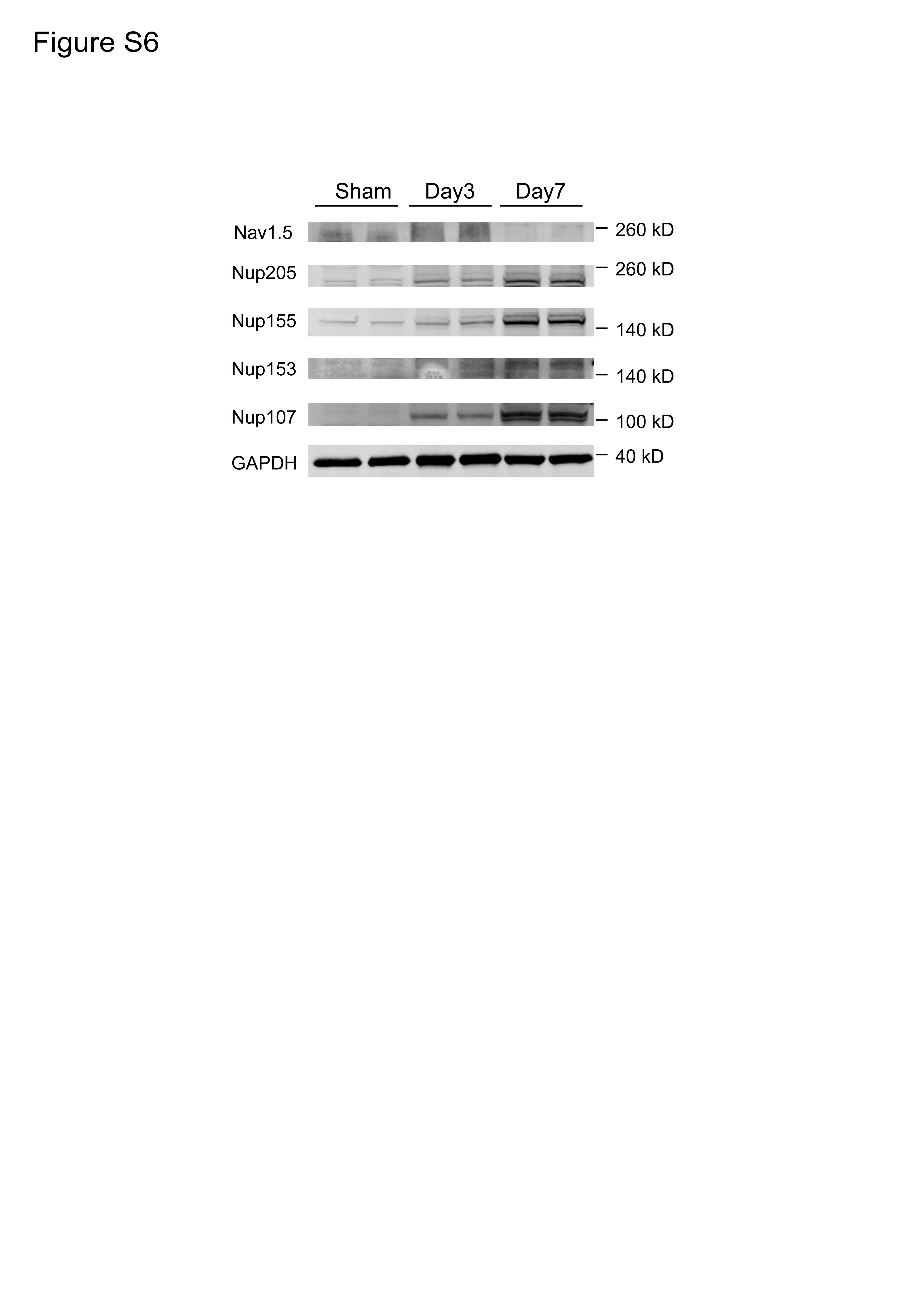

Supplement: Supplementary file 6 [file JCMM-23-1448-s006.tif]

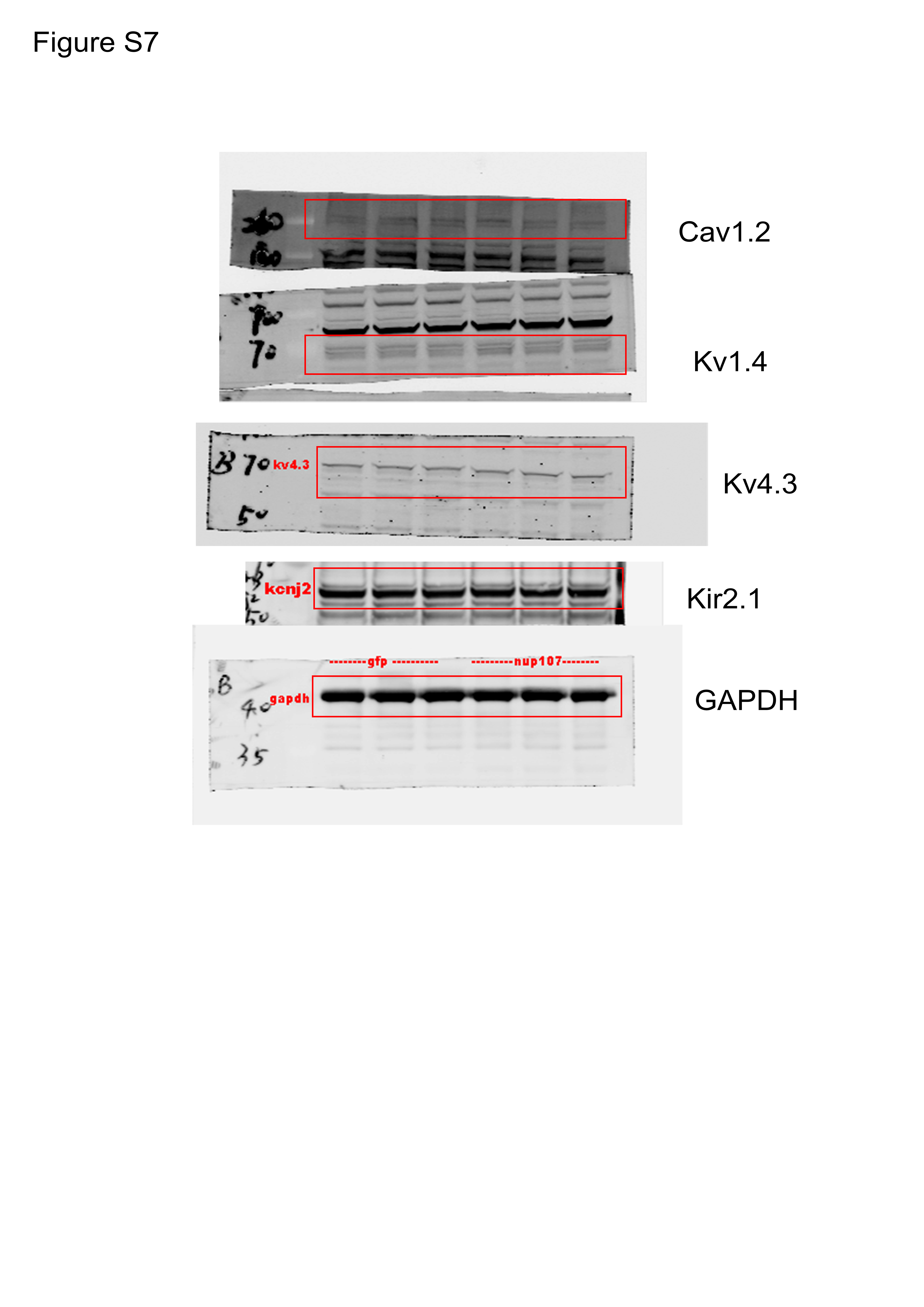

Supplement: Supplementary file 7 [file JCMM-23-1448-s007.tif]

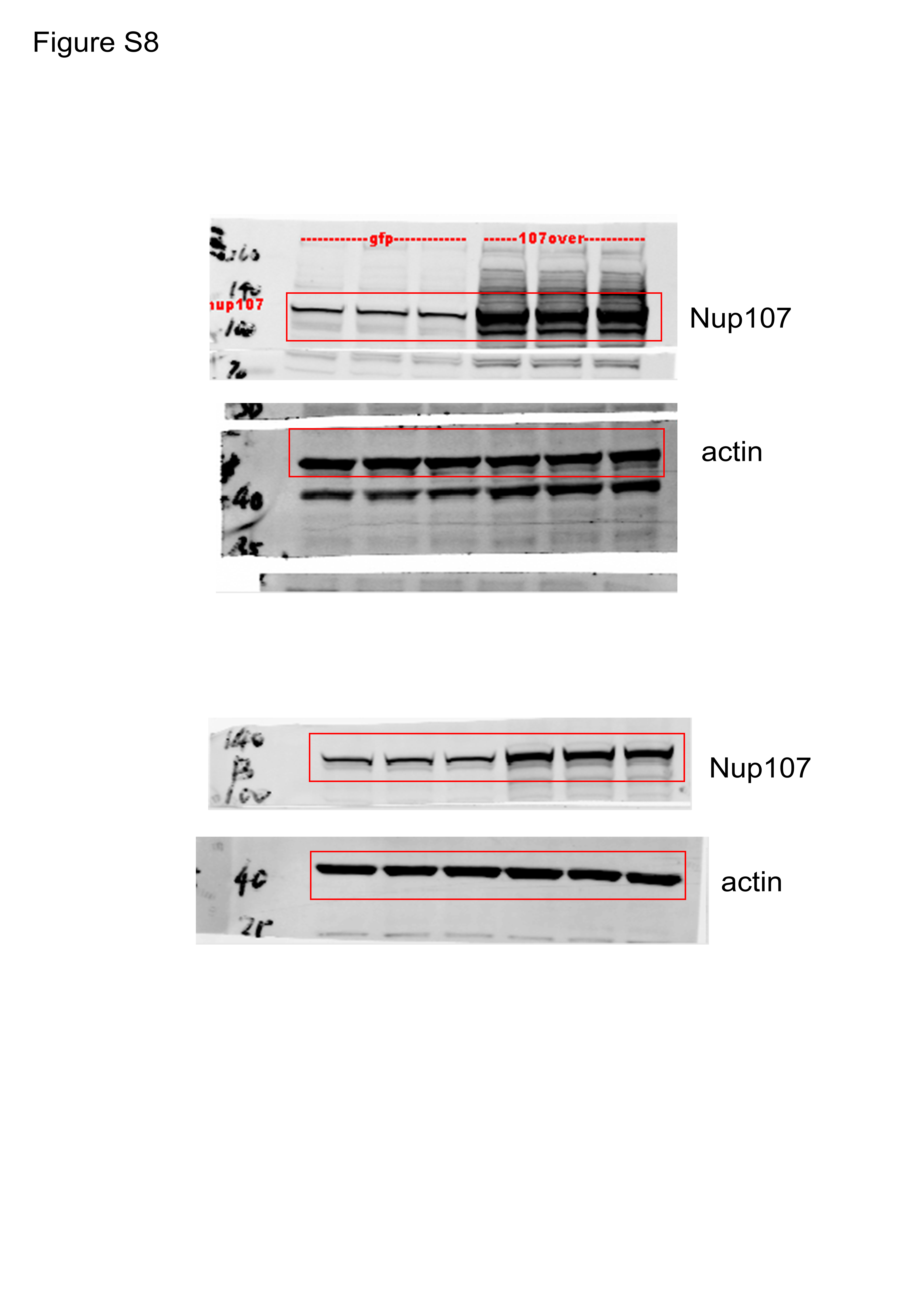

Supplement: Supplementary file 8 [file JCMM-23-1448-s008.tif]

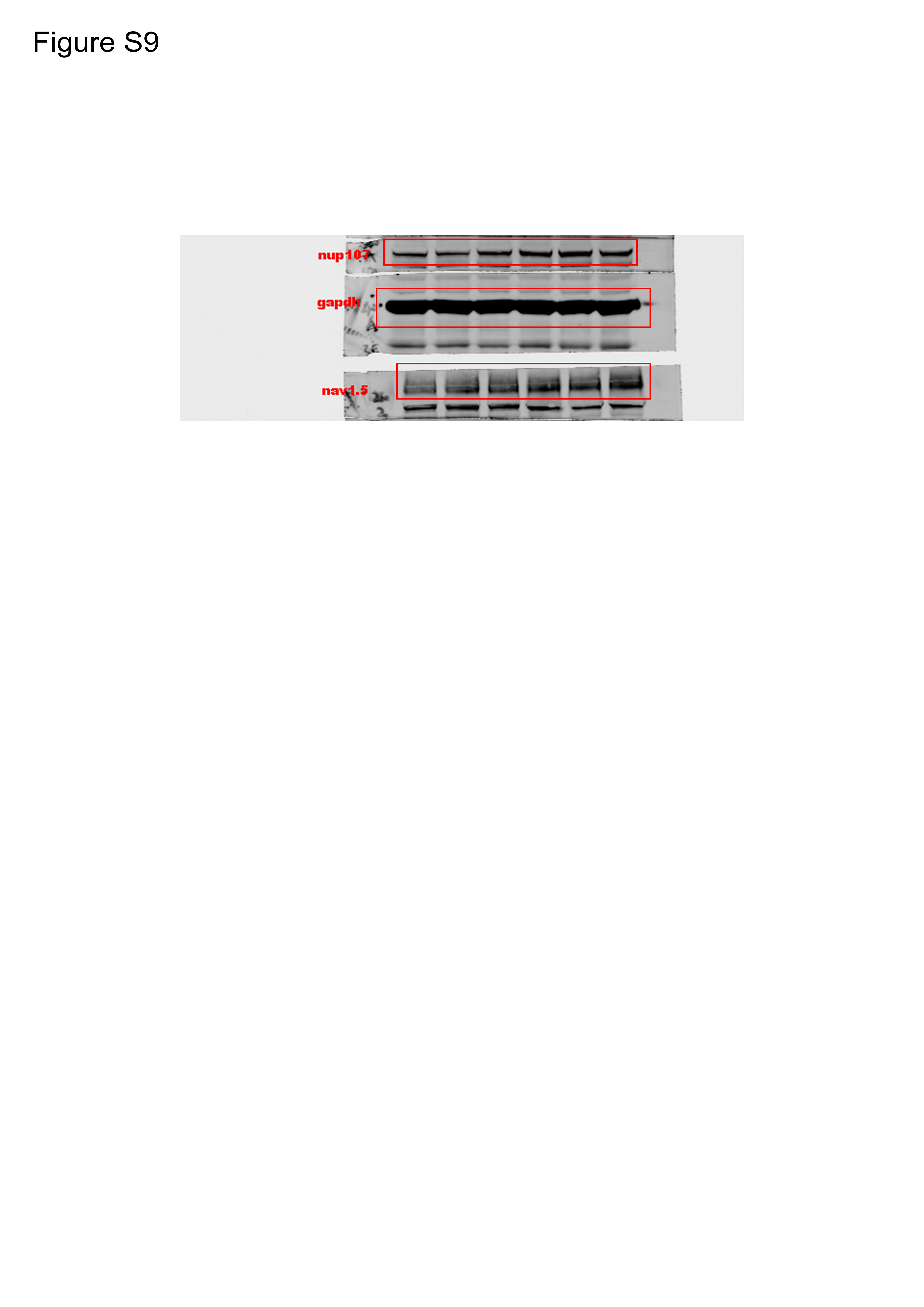

Supplement: Supplementary file 9 [file JCMM-23-1448-s009.tif]
