# Supplementary material for: Nucleoporin 107 facilitates the nuclear export of Scn5a mRNA to regulate cardiac bioelectricity
Source: J Cell Mol Med. 2018 Dec 3;23(2):1448–57. doi: 10.1111/jcmm.14051 (PMC6349201; doi:10.1111/jcmm.14051)
Supplement: Supplementary file 10 [file JCMM-23-1448-s010.docx]

**SUPPLEMENTARY MATERIAL**

**Supplementary Table 1**

Primers for real-time PCR

| **Gene name** | **Forward Primer (5' to 3' )** | **Reverse Primer (5' to 3' )** |
| --- | --- | --- |
| **Rat-Cacna1c** | **TTATGGCCTTCAAACGTGGC** | **CGAAGGCCCGAATCATTGTG** |
| **Rat-Kcnd2** | **GCCTGGAGAAAACCACGAAC** | **GGGAGGGGCTGTGACTTGA** |
| **Rat-Kcnd3** | **CTCCAGCCGACAAGAACAAG** | **CGGGTCACGGTCAAAGAAGT** |
| **Rat-Kcnj2** | **TGTGTTACAGACGAGTGCCC** | **CAGAGTTTGCCGTCCCTCAT** |
| **Rat-Kcna4** | **CAGCAGCAGGCTATGTCCTT** | **TTCACGCATGCTGGCTCTTA** |
| **Rat-Scn5a-mature** | **GACTGCTGGGAACGCCTATA** | **ATGGTGGCTTGGTTTTGCTC** |
| **Rat-Scn5a-Int1** | **ACATACGCTTCACCTTCTATT** | **ACTGGCTCCCTGACCTTT** |
| **Rat-Scn5a-Int2** | **TCAAGAGTCGCCAAACCA** | **TAGCCACCAGGGCATTAG** |
| **Rat-Scn5a-Int3** | **ATATTGAGACCCTGTCCTG** | **TTTACCTCCCTCTGTTCG** |
| **Rat-Scn5a-Junc1** | **CTACCCTGCTATTTGTCTGTG** | **CCATCTTCTCATCCTGCTT** |
| **Rat-Scn5a-Junc2** | **CCATGACCAAACACCCTT** | **GAAGATAGTCGTTTCCTCCT** |
| **Rat-Scn5a-Junc3** | **AGTTGTACTGGACCAAGGTG** | **CTCTGAATGGAGGAAGGAA** |
| **Rat-Gapdh** | **CTGGTGCTGAGTATGTCGTGGA** | **AGTTGGTGGTGCAGGATGCATT** |
| **Rat-β-actin** | **CCCATCTATGAGGGTTACGC** | **TTTAATGTCACGCACGATTTC** |
| **Rat-18S** | **GTTGAACCCCATTCGTGAT** | **GCTTATGACCCGCACTTACT** |
| **Rat-β-tubulin** | **AGGAAATAACTGGGCAAAGG** | **TGATGCGGTCTGGGTACTCTT** |
| **Rat-actinin** | **CAAACCCGATGAAAGAGCCA** | **GCGTCGAATCCATTCCAAGA** |
| **Rat-Scn5a-Long** | **ACCACGGATGTGTTACTATGTGG** | **GTTGGTTACTGGGGCCAAAG** |
| **Rat-Gapdh-Long** | **CAAGTTCAACGGCACAGTCA** | **TGTCATCATACTTGGCAGGT** |
| **Rat--β-actin-Long** | **CCCAGATCATGTTTGAGACC** | **GCTAGGAGCCAGGGCAGTAA** |
| **Rat-actinin-Long** | **CCTTGGACTCTGTGCCCTCA** | **ATTCCTCATAGCCCTTCTCG** |
| **Rat-Scn5a-Rip** | **ATTGTGCCCTGACGAACC** | **ACCCGGCTACTAGGGTGT** |
| **Rat-Scn5a-5’UTR** | **CCGTGGGATGCGGGGATCGC** | **CGGCGTAGGGCTTGGGGCAG** |
| **Rat-Scn5a-CDS-1** | **TGAAGGCAGGTGAGAACCCA** | **AGGAGCCCAGAAAGATGACG** |
| **Rat-Scn5a-CDS-2** | **CCGCTGGCACATGATGGACT** | **GGTTGTCTGCGCTGAAGGAG** |
| **Rat-Scn5a-CDS-3** | **AGCAAACAGGAATCCCAAGT** | **AACTGTCCTCAGGGGTCTCA** |
| **Rat-Scn5a-CDS-4** | **GGGGACCTGCCTCTGAACTA** | **CTGCTCCTCATACCCTCTGG** |
| **Rat-Scn5a-CDS-5** | **TCTTCGACTTTGTGGTGGTC** | **AAGAGGGCGGGCAGGGACAT** |
| **Rat-Scn5a-CDS-6** | **CGCCAAACCCAACCAGATAA** | **CTCCTCAGGGTGGTGGTGAT** |
| **Rat-Scn5a-3’UTR-1** | **TGAGGTGGAATGGGAAAC** | **CTTGCTCAGGGTTAAGGAG** |
| **Rat-Scn5a-3’UTR-2** | **ACATGCTAGGGCGGGACA** | **AGGTGAAGCCTGACTAGAAAA** |
| **Rat-Scn5a-3’UTR-3** | **TTCTGCCCTTCCTTCCTC** | **CTCTTCCCTCAGCCTTTG** |
| **Rat-Scn5a-3’UTR-4** | **CCACTGGGACTCCAACTC** | **AGAAGACGCCTGGGATAG** |
| **Mouse-Gapdh** | **AGGTCAATGAAGGGGTCGTT** | **AAATGGTGAAGGTCGGTGTG** |
| **Mouse-Nup35** | **CCACTCCCGCTGAGTTTTGA** | **TTGATCGAGGTTGCGGAGTC** |
| **Mouse-Nup37** | **AGCTCTTCCTTGTGACCTCG** | **ATCTTCGCAATCCACGGTGT** |
| **Mouse-Nup43** | **ACAGCAGTACACTCCATGCC** | **GCCACCGGTAGCTACAACAT** |
| **Mouse-Nup50** | **TTGGGCTCGTTAAGCTCTGG** | **CTTGGGTGGCTCGTCATTCT** |
| **Mouse-Nup54** | **TCTACTACCTCGGCGACTCC** | **TGTTGGGGCTGAAAAGCTGA** |
| **Mouse-Nup62** | **GGTGTGAGCGGAGAGTGAAG** | **CAAATGTAAAGCCGCCAGCA** |
| **Mouse-Nup85** | **CTTTGGCAGCTGTCTGTGAG** | **ATCCAAGTTACTGCGGGCTC** |
| **Mouse-Nup88** | **GTCGAGAGGAAAGGGTTTCTCA** | **CGTTGCCTAGATGTCGCAGT** |
| **Mouse-Nup93** | **CCGTGCACCCCATTTAAGAG** | **GTCACTGCAGCTCTCCTACC** |
| **Mouse-Nup98** | **GGCCCTATTCATGGGACGTT** | **GGTGCTGACCTTGGGTTTCT** |
| **Mouse-Nup107** | **ATGGACAGGAGTGGCTTCGG** | **GGGTGTCTCAGTAGGCTTCG** |
| **Mouse-Nup133** | **CATGTTTCCGAGCGTCTCCT** | **ATCCCAGAGACAGACCCCTC** |
| **Mouse-Nup153** | **ACAGATGACCTCTCTGGGCA** | **TCACAAGGAGCACTTCGCTT** |
| **Mouse-Nup155** | **CAGCGTAGCATTGAGCTCGT** | **AAGGCACGCCGATTTCATTC** |
| **Mouse-Nup160** | **TGACACTAACAACAAGTTCCCCA** | **GAGCGTTGGCGTGTAAGTTG** |
| **Mouse-Nup188** | **TGGCAGCGGTTGTTAGAAGG** | **CAGCTCCTTCAGAGGTGACG** |
| **Mouse-Nup205** | **GTTGGCGGTAAATTCGGCTG** | **GGATCGCAACACCCTCTGAA** |
| **Mouse-Nup210** | **ATCTCCGGCTGCTTTCAACA** | **CCCTGCACATTGCCTAGTGA** |
| **Mouse-Nup214** | **AGTGGCATTCTGAGTAGAGCC** | **AGAACTGGCTGTGAAGGAAGG** |
| **Mouse-Ndc1** | **GGTTGAGTCTGTTCAGGCGA** | **AAAACGCGCCAAAGGATGTC** |
| **Mouse-Rae1** | **ACTTGCCGTCTCTCCATTCAG** | **GGCTATTTCCTGAGGAGTTGGG** |
| **Mouse-NupL1** | **TTCGGCTCAGGTATTGGCAC** | **CAGTGACTGCTCAGAACTGGAT** |
| **Mouse-NupL2** | **GCAGAGGCTTTGGGTCATCT** | **AACAGAGTTTAGCTGAAATATTGGG** |
| **Mouse-DDx19a** | **AGCATCTCTGCAGGTCGGC** | **TCATCGACTTGACAGCGGC** |
| **Mouse-Pom121** | **GCCTCGCGCTCTACCTG** | **ACCGTTGACTGGTGACTTGG** |
| **Mouse-Aaas** | CCAGATGGAAACCGACTGCT | AGACGTTCACCACTTGGGTC |

**Supplementary figure legends**

**Figure S1. The mRNA levels of nucleoporins in ventricular tissues from myocardial infarction in mice.**

(A) The mRNA levels of nucleoporin genes between the sham and myocardial infarcted ventricle tissues in mice, were estimated by RT-PCR.. *, P < 0.05. (B) Data mining from GSE83350 showing the mRNA levels of nucleoporin genes between the sham and myocardial infarcted ventricle tissues in mice.

**Figure S2. Overexpression of Nup107 in NRVMs by adenovirus.**

(A) Fluorescence imaging of NRVMs infected with Ad-Nup107 or Ad-GFP. Infected cells, green; nucleus, blue. Scale bar: 100 µm.

(B, C) Western blotting analysis showing that the Nup107 was markedly increased.

**Figure S3. Schematic diagram of Nup107 truncated mutants.**

Human Nup107 contains three domains, including the N-terminus, the C-terminus and a conserved domain, which is homologous to Nup84p in yeasts. Different truncates of Nup107 were constructed as shown in the diagram.

**Figure S4. The paralleled elevation of Nup107 and Nav1.5 in NRVMs unpon oxidative insult.**

Protein expression levels of Nup107 and Nav1.5 in cultured NVRMs subjected to H_2_O_2_ insult (100 uM) for 48 h.

**Figure S5. The luminescence intensities under the overexpression of numerous nucleoporins.**

HEK293 cells overexpressed with the luciferase reporters containing the CDS of *Scn5a* mRNA, were co-transfected with different nucleoporins for 48 h, followed by the examination of the luminescence intensities.

**Figure S6. The protein expression of other nucleoporins in infarcted heart tissues.**

The protein expression of Nav1.5 and nucleoporins (Nup107, Nup153, Nup155, and Nup205) in ischemic ventricular tissues from the rat model of acute myocardial infarction.

**Figure S7-9. The raw blot images in the present figures.**

Figure S7, the raw blot images of Figure 1D; Figure S8, the raw blot images of Figure S2; Figure S9, the raw blot images of Figure 4D.
